# Feasibility, Acceptability, and Potential Efficacy of a Self-Guided Internet-Delivered Dialectical Behavior Therapy Intervention for Substance Use Disorders: Randomized Controlled Trial

**DOI:** 10.2196/50399

**Published:** 2024-01-16

**Authors:** Alexander R Daros, Timothy H Guimond, Christina Yager, Emma H Palermo, Chelsey R Wilks, Lena C Quilty

**Affiliations:** 1 Campbell Family Mental Health Research Institute Centre for Addiction and Mental Health Toronto, ON Canada; 2 Addictions Division Centre for Addiction and Mental Health Toronto, ON Canada; 3 Department of Psychiatry University of Toronto Toronto, ON Canada; 4 Penn Center for Mental Health Perelman School of Medicine University of Pennsylvania Philadelphia, PA United States; 5 Department of Psychological Science University of Missouri-St Louis St. Louis, MO United States

**Keywords:** depression, anxiety, emotion dysregulation, digital interventions, dialectical behavior therapy, substance use disorder, alcohol use disorder, randomized controlled trial, eHealth, mobile phone

## Abstract

**Background:**

People with alcohol and substance use disorders (SUDs) often have underlying difficulties in regulating emotions. Although dialectical behavioral therapy is effective for SUDs, it is often difficult to access. Self-guided, internet-delivered dialectical behavioral therapy (iDBT) allows for expanded availability, but few studies have rigorously evaluated it in individuals with SUDs.

**Objective:**

This study examines the feasibility, acceptability, and potential efficacy of an iDBT intervention in treatment-seeking adults with SUDs. We hypothesized that iDBT would be feasible, credible, acceptable, and engaging to people with SUDs. We also hypothesized that the immediate versus delayed iDBT group would show comparatively greater improvements and that both groups would show significant improvements over time.

**Methods:**

A 12-week, single-blinded, parallel-arm, randomized controlled trial was implemented, with assessments at baseline and at 4 (acute), 8, and 12 weeks (follow-up). A total of 72 community adults aged 18 to 64 years were randomized. The immediate group (n=38) received access to iDBT at baseline, and the delayed group (n=34) received access after 4 weeks. The intervention (*Pocket Skills* 2.0) was a self-guided iDBT via a website, with immediate access to all content, additional text and email reminders, and additional support meetings as requested. Our primary outcome was substance dependence, with secondary outcomes pertaining to feasibility, clinical outcomes, functional disability, and emotion dysregulation, among other measures. All outcomes were assessed using self-report questionnaires.

**Results:**

iDBT was perceived as a credible and acceptable treatment. In terms of feasibility, 94% (68/72) of the participants started iDBT, 13% (9/68) were early dropouts, 35% (24/68) used it for the recommended 8 days in the first month, and 50% (34/68) were still active 4 weeks later. On average, the participants used iDBT for 2 hours and 24 minutes across 10 separate days. In the acute period, no greater benefit was found for the immediate group on substance dependence, although we did find lower depression (b=−2.46; *P*=.02) and anxiety (b=−2.22; *P*=.02). At follow-up, there were greater benefits in terms of reduced alcohol (b=−2.00; *P*=.02) and nonalcoholic substance (b=−3.74; *P*=.01) consumption in the immediate access group. Both groups demonstrated improvements in substance dependence in the acute (b=−1.73; *P*<.001) and follow-up period (b=−2.09; *P*<.001). At follow-up, both groups reported reduced depression, anxiety, suicidal behaviors, emotional dysregulation, and functional disability.

**Conclusions:**

iDBT is a feasible and acceptable intervention for patients with SUDs, although methods for improving engagement are warranted. Although results did not support efficacy for the primary outcome at 4 weeks, findings support reductions in substance dependence and other mental health concerns at 12 weeks. Notwithstanding the limitations of this study, the results suggest the potential value of iDBT in the treatment of SUDs and other mental health conditions.

**Trial Registration:**

ClinicalTrials.gov NCT05094440; https://clinicaltrials.gov/show/NCT05094440

## Introduction

### Background

Alcohol and substance use disorders (SUDs) are the leading causes of death and disability worldwide [1,2]. These conditions are often chronic, leading to elevated risks of co-occurring medical and mental health conditions, involvement with the criminal justice system, and loss of workplace productivity [1-4]. In 2019, the past-year use of alcohol, cannabis, tobacco, and illicit substances was 77%, 21%, 14%, and 3.6%, respectively, in Canadians [5]. Increased consumption during the COVID-19 pandemic in Canada and around the world has been linked to greater substance-related harms and concurrent mental health symptoms, such as depression, anxiety, and hopelessness [6]. Various evidence-based psychological treatments are available for SUDs; however, the availability and demand for these services come at a time when internet and mobile delivery formats are being promoted in care pathways [7]. These formats hold considerable public health promise in reducing the burden associated with SUDs. For example, a recent systematic review highlighted that existing mobile interventions were effective and rated as acceptable by people with SUDs [8].

### Psychological Treatments for SUDs

Although pharmacological treatments exist for some substances (eg, alcohol and opioids), they have mixed evidence in treating other SUDs (eg, cannabis and stimulants [9]). Thus, psychological treatments remain a necessary therapeutic avenue for SUDs and may be particularly promising for those with multiple substance use concerns. Although psychological treatments vary greatly in their approach and theoretical framework, they tend to produce moderate effect size reductions in substance dependence [10,11]. To date, the greatest evidence supports cognitive behavioral and motivational enhancement approaches for treating SUDs.

SUDs rarely occur in isolation and often co-occur with depressive, anxiety, bipolar, and traumatic stressor disorders [12]. Psychological treatments are well suited to treat multiple conditions simultaneously when they incorporate a transdiagnostic focus or approach. There is growing consensus that people with SUDs, regardless of a specific substance, report higher difficulties in regulating their emotions compared with control samples and often use alcohol or other substances to cope with negative emotions [13]. More broadly, difficulties in emotion regulation appear to be a transdiagnostic risk factor underlying not only the development and course of SUDs but also depressive, anxiety, bipolar, and traumatic stressor disorders [14,15]. They also represent a promising treatment target, as emotion regulation skills tend to improve during psychological treatments for SUDs, along with more general improvements in self-efficacy and coping [16,17]. One psychological intervention that may be of substantial interest is dialectical behavior therapy (DBT), which was developed to treat individuals with high emotion dysregulation and includes comprehensive skills training in the domains of mindfulness, distress tolerance, emotion regulation, and interpersonal effectiveness.

DBT is a third-wave psychological intervention designed for patients with complex and severe behavioral, emotional, and interpersonal dysfunction [18,19]. DBT was first developed and found to be effective for severe clinical presentations related to suicidal behavior, nonsuicidal self-injury, and borderline personality disorder in adolescents and adults (refer to the study by Neacsiu et al [20] for review). Over time, DBT was reconceptualized as a transdiagnostic intervention appropriate for other mental health conditions and now includes specific content relevant to SUDs as well as other addictive behaviors [16,21,22]. Nevertheless, outpatient programs offering DBT are often safeguarded for those with acute suicide risk and behavioral problems. Importantly, although DBT was originally developed as a year-long multimodal intervention, evidence suggests that relatively brief formats focusing on DBT skills training (eg, 8-32 wk) are effective in treating SUDs, either as a primary condition or a co-occurring presentation in numerous clinical trials [16,22-24]. Despite these promising results, further research is needed to support the potential benefits of digital formats of DBT skills training, particularly within inclusive samples that reflect those seeking support for SUD.

### Internet-Delivered DBT

Another way to increase the availability of DBT is through internet and mobile delivery formats. Thus far, research on internet-delivered DBT (iDBT) has been promising. In a review of 11 studies, iDBT was feasible and effective, although these results were based on small sample sizes, and few studies adopted a more rigorous methodology (eg, randomized controlled trials [RCTs] [25]). Various methods have been used, such as therapist-led sessions delivered via web-based videoconferencing [26], asynchronous material delivered via email [27], self-guided stand-alone websites [28,29], and therapist-guided programs [30]. Studies that evaluated potential efficacy suggested that iDBT was at least as effective as control conditions (waitlist or face-to-face) and was accepted by users. However, web-based delivery is not without harm or adverse events. One large-scale trial comparing integrated care management and skills training (ie, 4 self-guided DBT skills) for those with suicidal ideation found that the latter condition led to an increased risk of self-harm [31]. A discussion of the study suggested that it faced, among other issues, an implementation failure [32]. Thus, these and other considerations should be incorporated in future work.

In a seminal study, Wilks et al [30] evaluated therapist-guided iDBT in a sample of participants who are suicidal and alcohol dependent in a completely remote manner. This 8-week waitlist-controlled RCT delivered video trainings on mindfulness (2 wk), addiction (2 wk), emotion regulation (3 wk), and distress tolerance (1 wk) using an e-learning web-based platform along with handouts and worksheets delivered via email. The content was developed in collaboration with the developer of DBT. The intervention produced significant reductions in suicidal ideation, alcohol consumption, and emotion dysregulation. Although the treatment was deemed safe and acceptable to participants, there was substantial dropout, and technical issues were reported as a barrier to adherence [33]. Nevertheless, those who remained in the study reported that it was useful.

Following this work, a more advanced iDBT intervention called *Pocket Skills* (version 1.0) was created to overcome the accessibility and engagement issues encountered previously [34]. It is available through an internet browser on any device (ie, computer, tablet, or smartphone) and offers an interactive experience by using a chatbot along with embedded video lessons and practice. *Pocket Skills* 1.0 was evaluated in a single-arm trial as an adjunct intervention in individuals with a range of mental disorders completing in-person DBT for 4 weeks. The results of the study were promising, with both quantitative and qualitative evidence for its feasibility, acceptability, and potential use as an adjunct. We developed this study based on these 2 previous studies.

### Current Study

This study aims to evaluate version 2.0 of *Pocket Skills* and advance the literature in several ways. First, the current investigation evaluates *Pocket Skills* 2.0, which includes some of the content from version 1.0, as well as revised and novel materials that have not yet been evaluated. Second, the delivery of iDBT in this study was predominantly self-guided, with limited therapist guidance compared with the previous trial that used iDBT intervention as a therapeutic adjunct [34]. Third, this investigation represented a more controlled study of *Pocket Skills* 2.0 as a stand-alone treatment in a sample of treatment-seeking adults with SUDs who were not receiving any other forms of psychological treatments. Finally, this investigation randomized participants to immediate versus delayed access to advance the previous single-arm study. A 12-week single-blinded parallel-arm waitlist-controlled RCT was initiated, with participants randomized to receive immediate access to the intervention or delayed access after 4 weeks. The 4-week intervention and follow-up periods are in line with previous implementations of self-guided digital mental health interventions [35-37]. These studies have found that attrition rates start to increase steadily after 4 weeks and especially after 7 to 8 weeks (eg, >50%).

Specifically, we hypothesized that greater than 50% of participants would start the intervention (H1a); not drop out early (H1b); engage with the intervention at a recommended dose of twice a week (or 8 d) in the first 4 weeks (H1c); and would still be using the intervention after 4 weeks (H1d). We also hypothesized that participants would rate the intervention as credible and acceptable on established measures (H1e). Second, we hypothesized that (H2a) participants in the immediate versus delayed iDBT group would show significantly greater improvements in our primary outcome of substance use dependence at the acute (week 4) and follow-up periods (week 12) in the form of an interaction effect (group×time). In addition, we hypothesized (H2b) significantly greater improvements for the immediate versus delayed iDBT group for our secondary outcomes (ie, depression, anxiety, emotion dysregulation, suicidality, functional disability, dispositional mindfulness, DBT skills, risky behaviors, and frequency of alcohol and substance use). Third, we hypothesized that iDBT would (H3) produce significant main effect improvements in both groups in the acute (week 4) and follow-up phases (week 12) of the intervention for all outcome measures.

## Methods

### Study Design

A 2-arm, single-blinded, parallel-group, preregistered RCT design was implemented, comparing individuals who received iDBT immediately with those who were first wait-listed for 4 weeks and then offered the intervention (delayed iDBT group). Assessments were completed at baseline and at 4 weeks, with additional follow-ups at 8 and 12 weeks. A CONSORT-EHEALTH (Consolidated Standards of Reporting Trials of Electronic and Mobile Health Applications and Online Telehealth) checklist was completed with more detailed information on the study design (Multimedia Appendix 1).

### Ethical Considerations

All study procedures were approved by the Centre for Addiction and Mental Health research ethics board (#016/2021), and this research complied with the Declaration of Helsinki of 1975, as revised in 2000. Data is stored in a de-identified format to safeguard participant information.

### Participants and Recruitment

Enrollment ran from August 2022 to March 2023, and all follow-ups were completed by June 2023. Participants were recruited from psychiatric hospital clinician referrals, waitlists, and research registries and from the surrounding community through several methods of advertisement (eg, hospital and other websites, social media posts, private DBT clinics, and local community organizations). All advertisements sought individuals who wanted to reduce their alcohol or substance use and specifically stated that they would be offered an internet-delivered intervention.

All prospective participants were initially informed about the study and were prescreened for eligibility over the phone. Inclusion criteria were as follows: (1) aged 18 to 65 years; (2) fluent in English; (3) understanding and willingness to comply with study requirements; (4) referred to addictions programming at our hospital or seeking treatment from the community, but not currently receiving any CBT or DBT intervention (support groups and psychiatric services were allowed); (5) alcohol or SUD in the past year; (6) use of alcohol or substance in the past month; (7) access to the internet (and assumed literacy); and (8) at least contemplation levels of wanting to reduce alcohol or substance use on the Contemplation Ladder measure [38]. Exclusion criteria included (1) any known practical factors that would preclude participation, (2) acute psychiatric (ie, suicidality, psychotic disorder) or medical condition (ie, acute intoxication or withdrawal) requiring medical attention, and (3) participation in another psychological intervention or treatment study. We did not exclude participants based on whether they were taking psychotropic medications or not.

### Registration

The trial was registered with the ClinicalTrials.gov database (NCT05094440) on October 14, 2021. A revised registration was published on September 6, 2022, in line with changes to our protocol between our pilot study and this study. In this study, our analysis focused on the measures included in registration. One modification of the registered protocol was made, that is, the addition of a DBT skills measure at all time points to permit the evaluation of how this intervention was linked to changes in this key treatment target. Feasibility, acceptability, and engagement metrics were decided a priori for study implementation and were included in our study-specific protocol.

### Randomization and Blinding

Participants were randomized to immediate or delayed iDBT using a blinded envelope system to ensure allocation concealment, with 3 randomization blocks (4, 6, and 8 participants). The randomization procedure was blinded to the participants and the experimenter who ran all baseline sessions (ARD). Thus, neither party knew which group the participant would be allocated to until after the informed consent and baseline procedures were completed. None of the participants withdrew immediately following randomization. The experimenters were not blinded to the procedures following the baseline session, including the follow-up assessments and contact. All follow-up assessments were conducted remotely and consisted solely of self-report measures.

### Procedure

Eligible participants attended a 45-minute baseline session via a videoconference, where they provided informed consent (electronically), completed a demographic questionnaire and semistructured diagnostic interview, and were randomized into either immediate or delayed access groups. At the end of the baseline session, those randomized to the immediate group were provided the iDBT website URL and an invitation code and completed the sign-in procedure (15 min) with the experimenter during the videoconference call. Those randomized to the delayed access group were scheduled for an additional appointment in 4 weeks, where they met with the experimenter again and completed the sign-in procedure (15 min). Thus, although the time spent with the experimenter was approximately the same, the delayed group met with the experimenter via videoconference twice. Each participant was sent a guide to the intervention via email, with a suggested 8-week protocol.

Follow-up questionnaires, completed via REDCap (Research Electronic Data Capture; Vanderbilt University), were automatically distributed via email or text every 4 weeks. Text and email reminders for the follow-up questionnaires were sent daily for up to 4 days until completed, starting 2 days before each assessment was due. To support engagement, additional text messages were sent to consenting participants (56/72, 78%) twice a week for the first 4 weeks following the start of iDBT in both groups (following this point, reminders were discontinued). These text messages contained a link to a short REDCap survey that encouraged use, queried whether participants wanted a follow-up call, and reported any technical issues. Participants could request additional calls or meetings with the experimenter (via REDCap survey or email) to troubleshoot or clarify different components of the website; however, <10 of these calls or meetings took place throughout the study. Participants were compensated up to CAD $70 (US $45.5) for the completion of these procedures (CAD $10 [US $6.5] for baseline and CAD $20 [US $13] each for the 4-, 8-, and 12-week assessments). On average, participants were compensated CAD $59 (US $38.35), including those who did not collect their final payment.

### Intervention

**Figure 1:**
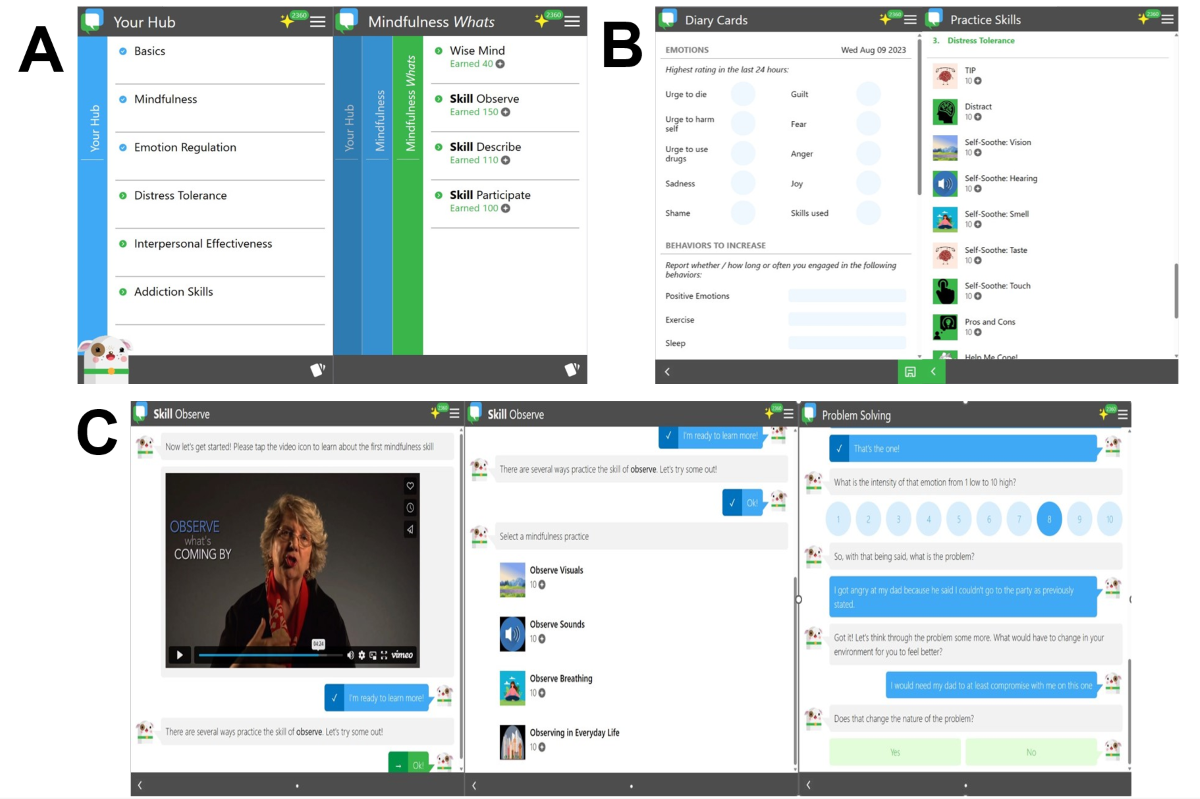
Screenshots depicting different features of the Pocket Skills 2.0 internet-delivered dialectical behavior therapy intervention: (A) displays the main Your Hub page, with the next screen showing the submenu selection within the Mindfulness module; (B) shows the optional Diary Card page to input various skills training targets, with the next screen showing the Practice skills page with quicker access to skills training without lessons; and (C) shows the initial portion of the Mindfulness Observe skill lesson, with an embedded video featuring Dr Linehan. The second screen shows the chatbot initializing an interactive skills training exercise, and the third screen shows the types of open- and closed-ended response options along with an example Likert-type rating scale.

*Pocket Skills* 2.0 is an iDBT intervention developed by author CRW in collaboration with Microsoft Research and Dr Marsha Linehan; it is built upon the most recent DBT manual available [18]. It uses a web-based portal built on the Microsoft Azure platform that is compatible with any internet browser in addition to the Android and iOS mobile operating systems. This iDBT intervention incorporates lessons following the core modules of DBT as well as a specific module focused on addiction (Table 1 provides more details, and Figure 1 provides the screenshots). Within each module, participants selected a specific skill and were presented with a brief video featuring Dr Linehan introducing the skill and its uses. A practice session then ensues with the rule-based chatbot, which allows for feedback through both open-ended text input and a closed selection of responses. The chatbot guides users on how to select skills to use in different situations that may arise as well as the ability to gain points and unlock additional content, which increases user engagement. After logging in for the first time, participants were prompted to complete an introductory module in which they entered a nickname and set personal goals. Following the completion of this module, participants were able to enter any of the 5 DBT modules offered freely, without the need to unlock any content. The procedure in which iDBT was delivered in this study differs from that in previous studies (refer to Multimedia Appendix 2 [30,34] for a comparison).

**Table 1:** List of skills covered within the Pocket Skills 2.0 internet-delivered dialectical behavior therapy (DBT) intervention.

Module	DBT skills	Brief training description
Mindfulness	Introduction to mindfulness; wise mind; observing, describing, and participating; and nonjudgment, one-mindfully, and effectively	Introduces the foundational skills to develop nonjudgmental awareness of the present and practice mindfulness with skillful effectiveness.
Emotion regulation	Introduction to emotion regulation; understanding emotions; check the facts, opposite action, and problem-solving; accumulating positives and pleasant events; and building mastery and coping ahead	Teaches the functions of emotions, how to describe them, and skills to reduce the frequency and quantity of unwanted emotions. Also teaches skills to build resilience against future negative emotions.
Distress tolerance	Introduction to distress tolerance; TIP^a^, distraction (ACCEPTS^b^), and self-soothe; pros and cons; and Help Me Cope!	Teaches skills to weather crises and intense negative emotions, manage experiential changes, and produce emotional and cognitive change. Help Me Cope! helps the user pick a coping strategy based on a few contextual questions.
Interpersonal effectiveness	Introduction to interpersonal effectiveness; DEARMAN^c^, GIVE^d^, and FAST^e^; and Dime Game	Teaches skills to navigate interpersonal situations and needs more effectively. Dime Game helps the user evaluate a situation for how firmly to make a request or say no.
Addiction	Introduction to addiction; pros and cons (addiction context); dialectical abstinence and clear mind; and community reinforcement and burning bridges	Helps learners find a middle path between sobriety and unrestrained substance use. Helps learners develop a clear mind and other strategies to stop or reduce problematic substance use.

^a^TIP: temperature, intense exercise, and paced breathing.

^b^ACCEPTS: activities, contributing, comparisons, emotions, pushing away, thoughts, and sensations.

^c^DEARMAN: describe, express, assert, reinforce, be mindful, appear confident, and negotiate.

^d^GIVE: be gentle, act interested, validate, and use an easy manner.

^e^FAST: be fair, no apologies, stick to values, and be truthful.

### Measures

#### Diagnostic Interviews

The Diagnostic Assessment and Research Tool version 4.0 [39] was used to assess depressive, anxiety, bipolar, obsessive-compulsive, trauma and stressor, alcohol, and SUDs according to the *Diagnostic and Statistical Manual of Mental Disorders, Fifth edition* [40]. We also screened the presence of psychotic disorders. All interviews were completed by the first author, who is a licensed clinical psychologist.

#### Feasibility and Credibility Measures

For feasibility, we calculated the proportion of randomized participants who started the intervention (by signing in on the first day of access). Of those who started the intervention, we calculated the proportion that (1) dropped out of the intervention after starting (indicated by not logging in after the first day), (2) recorded at least 1 activity after 4 weeks, and (3) completed a recommended dose of using the intervention twice per week for the first month (8 d total). Next, we administered the 6-item Credibility and Expectancy Scale [41] at baseline to assess whether participants had favorable opinions of the intervention and its potential effectiveness before starting treatment. In line with previous work [42], the first 3 items were used to evaluate credibility (using a 9-point Likert scale), whereas a single item (item 4) was used to evaluate expectancy of clinical improvement (using an 11-point Likert scale ranging from 0% to 100%).

#### Acceptability and Engagement Measures

We used the 6-item Treatment Acceptability Questionnaire [43], which was administered at weeks 4, 8, and 12 to assess ratings of acceptability, perceived effectiveness, and trustworthiness using a 7-point Likert scale. In this analysis, we used only the week 4 and 12 scores. From the intervention source, we examined several metrics tied to engagement or use: the total amount of time spent on the website, the number of interactions with the website (eg, clicks, page views, and text inputs), unique days of log-in, and days of use spread. We then recalculated these metrics for the first 4 weeks, consistent with the acute period of the intervention.

#### Primary Outcome

The Substance Dependence Scale [44] is a 5-item self-report scale used to assess the severity of alcohol or substance dependence at the baseline and follow-up assessments. Higher scores indicated a higher level of substance dependence. Participants were first asked to indicate which class of substance (including alcohol) they were experiencing the most difficulties abstaining from, even if they reported no use in the past month. The ω reliability coefficient in this study was 0.95. All primary and secondary outcome measures were administered at each assessment point.

#### Secondary Outcomes

The Patient Health Questionnaire, Depression subscale [45], is a 9-item self-report measure used to assess depressive symptoms over the past 2 weeks, with excellent internal reliability and clinical utility in predicting depression. The ω reliability coefficient was 0.93.

The Generalized Anxiety Disorder-7 Scale [46] is a 7-item self-report measure used to assess generalized anxiety symptoms over the past 2 weeks, with excellent internal reliability and clinical utility in predicting generalized anxiety disorder. The ω reliability coefficient was 0.95.

The Suicidal Behaviors Questionnaire-Revised [47] is a 4-item measure of suicidal thoughts and attempts as well as future intent over the past month, with evidence for its reliability and clinical utility. The total score ranges from 3 to 18, with scores ≥8 indicating significant suicidal risk within clinical samples. The ω reliability coefficient was 0.87.

The World Health Organization Disability Assessment Schedule 2.0 [48] is a 12-item self-report measure assessing functional disability over the past month in several domains (cognition, mobility, self-care, and getting along with others). Higher scores indicate greater functional disability. The ω reliability coefficient was 0.94.

The Difficulties in Emotion Regulation Scale, Short Form [49], is a 16-item self-report measure with excellent internal consistency, assessing emotion dysregulation based on a 6-facet model first described by Gratz and Roemer [50]. Higher scores suggest greater emotion dysregulation difficulties. The ω reliability coefficient was 0.83.

The Mindful Attention Awareness Scale [51] is a 15-item self-report measure of dispositional mindfulness in the form of open or receptive awareness and attention to what is taking place in the present over the past month. Higher scores, which were summed and then averaged, reflected higher levels of dispositional mindfulness. Owing to an administrative error, the anchors were reversed when presented to participants for the entire duration of the study. Therefore, we reversed all scores to ensure a standard interpretation as above. The ω reliability coefficient was 0.94.

The DBT Ways of Coping Checklist [52] is a 59-item self-report measure that assesses the frequency of maladaptive and adaptive skills used to manage difficult situations over the past month, with good internal consistency and test-retest reliability. In this study, we only used the 38-item adaptive skills subscale, which includes skillful behaviors often learned in DBT without using DBT-specific language. The ω reliability coefficient was 0.80.

The National Institutes of Drug Abuse–modified Alcohol, Smoking, and Substance Involvement Screening Test is an adaptation of the original measure [53] used to assess alcohol, smoking, and substance use involvement. This measure was used to assess tobacco, cannabis, cocaine, amphetamine-type stimulants, inhalants, sedatives or sleeping pills, hallucinogens, and opioids. Each class of substance was rated for frequency over the past month using an ordinal scale: 0=never; 1=once or twice; 2=3 or 4 times; 3=5, 6, or 7 times; 4=2 or 3 times a week; 5=4 or 5 times a week; and 6=daily or almost daily. The ω reliability coefficient was 0.23, likely because of the heterogeneity and range of substances used in our sample.

The Daily Drinking Questionnaire [54] was used to assess the frequency of alcohol use on each day of a typical week. Participants were asked how many standard drinks they had consumed on a typical Monday in the past month, with separate questions for each day of the week. Responses were recoded into an ordinal scale: 0=none, 1=1 to 2 standard drinks, 2=3 to 4 standard drinks, 3=5 to 7 standard drinks, 4=8 to 10 standard drinks, 5=11 to 14 standard drinks; and 6=≥15 standard drinks. The ω reliability coefficient was 0.96.

The Risky, Impulsive, and Self-Destructive Questionnaire [55] is an inventory of 38 risky, impulsive, and self-destructive behaviors that sometimes cause problems for people. For brevity and to avoid overlap with other measures, we only used the 4-item *risky sexual behavior* subscale and the 4-item *reckless behavior* subscale. We recoded the frequency of responses, which were evaluated over the past month, into an ordinal scale: 1=none, 2=once or twice, 3=3 to 4 times, 4=5 to 6 times, 5=7 to 9 times, and 6=≥10 times. The ω reliability coefficient was 0.86.

### Statistical Analysis

#### Overview

No outcome measure data were missing from the baseline, and participants returned at least partially completed follow-up questionnaires at rates of 94% (68/72; week 4), 78% (56/72; week 8), and 81% (58/72; week 12). At follow-up, scores for outcome measures were only used if there were <10% of items missing, and we treated outcome measures with no data as missing. The frequency of nonalcoholic substance use, standard alcoholic drinks per day, and risky impulsive behaviors was first recoded using ordinal values to approximately equate each scale with respect to their frequency of occurrence. For each measure, we took the average of each ordinal item score and then rounded the average value to the nearest one to serve as the dependent variable. This rounding was required as an ordinal regression relies on categorizing each value of the ordinal dependent variable as a factor variable. There are several ways to analyze ordinal variables, and this procedure was supported by our biostatistical consultation team.

Descriptive statistics were used to evaluate treatment feasibility, acceptability, and engagement data. Chi-square test, Fisher exact test, and 2-tailed *t* test analyses were used to evaluate baseline differences. Engagement data consisted of time stamped logs of each interaction (ie, clicks, page views, and text inputs) with the website, organized hierarchically within persons, with a total of 39,884 observations. To capture the time spent on iDBT, we ordered the data in Excel (Microsoft Corporation) according to time within persons and calculated a difference score (delta time) between rows. This difference score assessed the time between one meaningful interaction and the next. We then applied a filter to remove any difference scores >30 minutes to account for participants taking breaks or not returning to the app until the next day, capturing 93% of the data. A 10-minute filter captured 92% of the data; however, we wanted to account for playing video content, which could run up to 10 minutes, and the potential of practicing skills live, while remaining on 1 of the web pages. Once the filter was applied, we also calculated the number (and spread) of dates the app was used as well as the number of observations per person, which we called the meaningful interactions calculation. Sensitivity analyses were then performed by examining the same metrics over the first 4 weeks and the time spent on each iDBT module.

All other statistics were run in the statistical program R (version 4.2.1; R Foundation for Statistical Computing). To evaluate the internal consistency of our measures over time, we calculated the between-person ω reliability coefficient [56] statistic using the *omegaSEM* function from the *MultilevelTools* package (version 0.1.1). To characterize changes over time for our continuous variables, we ran a series of linear mixed models with the *lme4* package (version 1.1-26 [57]), with each primary and secondary outcome serving as a dependent variable in separate models. To characterize changes over time for our ordinal variables, we ran additional linear mixed cumulative link models using the *ordinal* package (version 2022.11-16) with separate models for each outcome. As per recommendations, we adjusted each model by incorporating the baseline dependent variable value for each person irrespective of whether the difference was significant between groups [58].

All models included a random intercept for a person and relied on restricted maximum likelihood estimation. We omitted any random slope effects throughout the analyses because all our independent variables were level-2 grouping variables.

Each primary and secondary outcome variable was assessed with models containing an interaction effect (group×time, as factor variables) and main effects only (group+time, as factor variables) along with a continuous covariate controlling for the baseline assessment of each outcome per person. The final model chosen for interpretation was the better fitting model based on lower Akaike information criterion and Bayes information criterion values. Therefore, if the model fit was improved by the inclusion of the interaction term, we report that model; otherwise, we removed the interaction term and report the model with the main effects only. All model comparisons were evaluated using maximum likelihood estimation with the *lmerTest* (version 3.1-3 [59]) package, which uses the Satterthwaite *df* method. To further reduce the number of statistical tests reported, we also opted to interpret only the week 4 and week 12 contrasts against baseline as these were the most pertinent time points to address our hypotheses. Our α significance level was *P*=.05, and all statistical tests were 2 tailed. The outputs of each final statistical model are provided in Multimedia Appendix 2 for full transparency.

#### Power

To achieve at least a medium effect size reduction in substance dependence, as suggested by Wilks et al [30], we would require a minimum sample of 60 as per G*Power (version 3.1.9.7; Cohen *f*=0.15; 2 groups; 4 measurements over 12 wk; power=0.95; α=.05; and correlation between measures of at least 0.70) [60]. With an expected attrition rate of approximately 20%, we aimed to recruit approximately 72 to 75 individuals in total.

## Results

### Hypothesis 1: Feasibility and Acceptability

#### Participant Enrollment and Demographic Characteristics

Initially, 116 individuals were assessed for eligibility, and 72 participants aged 18 to 64 years completed all baseline procedures and were randomized. Demographic and clinical characteristics of the sample are presented in Table 2; these characteristics did not differ between groups, suggesting that the randomization procedure was successful. Of the 72 participants, 9 (13%) met the full threshold criteria for >1 SUD. The primary nonalcohol substance disorder across the sample was cannabis (22/72, 31%); nicotine (10/72, 14%); stimulants (7/72, 10%); and sedative, hypnotic, or anxiolytic (1/72, 1%). None of the participants had a current opioid use disorder. Participants met the criteria for a median of 3 psychiatric diagnoses overall (mean 3.30, SD 1.69; range 1-7).

**Figure 2:**
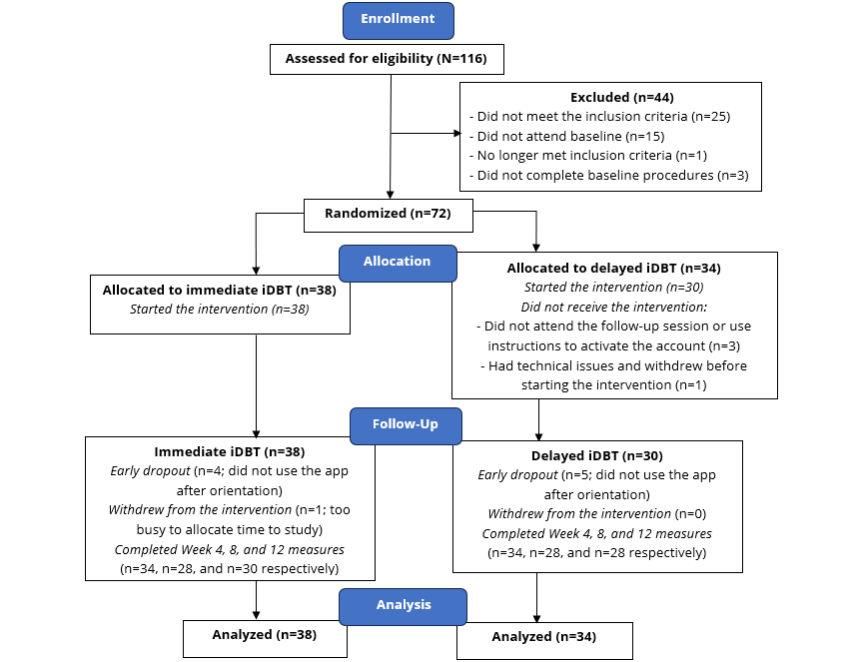
CONSORT (Consolidated Standards of Reporting Trials) diagram depicting the participant flow through the study. iDBT: internet-delivered dialectical behavior therapy.

At baseline, 46% (33/72) of the participants reported taking psychotropic medications in the past month, 22% (16/72) of the participants reported seeing a psychiatrist in the past month, and 8% (6/72) of the participants reported attending a community resource (eg, Alcoholics Anonymous and peer support group) in the past month. These rates did not increase when the participants reported the same services at each follow-up. As we did not restrict new options for care following baseline, 4 participants reported having access to outpatient programing at week 4, but only 2 reported this at both weeks 8 and 12. Figure 2 summarizes the study flow of participants in a CONSORT (Consolidated Standards of Reporting Trials) diagram.

**Table 2:** Demographic characteristics of the total intent-to-treat sample and by condition, with statistical comparisons (N=72).

Characteristics			Total ITT^a^ (N=72)		Immediate iDBT^b^ (n=38)		Delayed iDBT (n=34)		Group comparison statistical value			Group comparison *P* value	
Age (y), mean (SD)			34.1 (11.9)		33.4 (10.5)		34.8 (13.3)		t_70_*=*0.50			.62	
**Sex, n (%)**										χ^2^_2_=0.2			.69
	Female	47 (65)		24 (63)		23 (68)		—^c^			—		
	Male	25 (35)		14 (37)		11 (32)		—			—		
	Other	0 (0)		0 (0)		0 (0)		—			—		
**Gender,n (%)^d^**										χ^2^_2_=0.5			.77
	Woman	43 (60)		22 (58)		21 (62)		—			—		
	Man	24 (33)		13 (34)		11 (32)		—			—		
	Other (nonbinary, transgender, genderfluid, or other)	6 (8)		4 (10)		2 (6)		—			—		
**Sexual orientation, n (%)^d^**										χ^2^_3_=1.4			.70
	Heterosexual	44 (61)		22 (58)		22 (65)		—			—		
	Lesbian or gay	5 (7)		3 (8)		2 (6)		—			—		
	Bisexual	11 (15)		5 (13)		6 (18)		—			—		
	Other (pansexual, queer, asexual, questioning or not sure, or prefer not to answer)	12 (17)		8 (21)		4 (12)		—			—		
**Race or ethnicity, n (%)^d^**										χ^2^_5_=4.8			.45
	Black (African, North American, and Caribbean)	7 (10)		2 (5)		5 (15)		—			—		
	East or Southeast Asian	5 (7)		4 (10)		1 (3)		—			—		
	Latin American	5 (7)		3 (8)		2 (6)		—			—		
	South Asian	9 (12)		5 (13)		4 (12)		—			—		
	White	48 (67)		25 (66)		23 (68)		—			—		
	Other (First Nations, Middle Eastern, mixed, or not listed)	8 (11)		6 (16)		2 (6)		—			—		
**Marital status, n (%)**										χ^2^_4_=4.9			.18
	Single	39 (54)		25 (66)		14 (41)		—			—		
	Dating	18 (25)		8 (21)		10 (29)		—			—		
	Married	10 (14)		3 (8)		7 (21)		—			—		
	Other (divorced, widowed, or separated)	5 (7)		2 (5)		3 (9)		—			—		

**Employment status, n (%)**										χ^2^_5_=1.5			.83
	Full-time employed	29 (40)		17 (45)		12 (35)		—			—		
	Part-time employed	19 (26)		10 (26)		9 (27)		—			—		
	Unemployed	14 (19)		6 (16)		8 (23)		—			—		
	On disability	7 (10)		3 (78)		4 (12)		—			—		
	Prefer not to say	3 (4)		2 (5)		1 (3)		—			—		
**Current conditions, n (%)**													
	Major depressive disorder	37 (51)		20 (53)		17 (50)		Fisher exact test			.99		
	Persistent depressive disorder	18 (25)		10 (26)		8 (23)		Fisher exact test			.99		
	Bipolar I or II disorder	6 (8)		4 (10)		2 (6)		Fisher exact test			.68		
	Generalized anxiety disorder	37 (51)		21 (55)		16 (47)		Fisher exact test			.64		
	Social anxiety disorder	22 (31)		10 (26)		12 (35)		Fisher exact test			.45		
	Posttraumatic stress disorder	18 (25)		10 (26)		8 (23)		Fisher exact test			.99		
	Other anxiety disorder	11 (15)		8 (21)		3 (9)		Fisher exact test			.20		
	Alcohol use disorder	47 (65)		23 (60)		24 (71)		Fisher exact test			.46		
	Any substance use disorder	40 (56)		24 (63)		16 (47)		Fisher exact test			.24		
	Cannabis use disorder	24 (33)		12 (32)		12 (35)		Fisher exact test			.81		
	Nicotine use disorder	15 (21)		9 (24)		6 (18)		Fisher exact test			.57		
	Stimulant use disorder	9 (12)		7 (18)		2 (6)		Fisher exact test			.16		
	SH or A^e^ use disorder	1 (1)		1 (3)		0 (0)		Fisher exact test			.99		

^a^ITT: intent-to-treat (ie, completed baseline procedures and randomized to condition).

^b^iDBT: internet-delivered dialectical behavioral therapy.

^c^Some cells are empty because we report the group comparison statistic for the overall category above.

^d^Participants could select multiple options.

^e^SH or A: sedative, hypnotic, or anxiolytic.

#### Feasibility and Credibility

Moreover, 94% (68/72) of the randomized participants started the intervention. Three participants in the delayed iDBT group did not attend the follow-up session and never connected to the intervention following attempts to reschedule and instructions provided via email. One additional participant withdrew from the delayed group owing to technical issues and being unable to sign in and therefore did not access the intervention. One participant asked to withdraw from the immediate group because of lack of time to spend on the intervention. In addition, 87% (59/68) of those who started iDBT used it for longer than the initial sign-in day (early dropouts: 9/68, 13%), 50% (34/68) recorded at least 1 iDBT activity after 4 weeks, and 35% (24/68) used it according to the recommended dose of 8 days within the first month. Finally, 63% (43/68) of the participants used it for at least 1 hour in total.

Perceptions of intervention credibility at baseline were positive (mean 74%, SD 16%; range 44%-100%). Participants thought it was logical and it was likely to raise their quality of functioning, and they were confident in recommending it to another person. Participants also estimated a mean 58% (SD 21%; range 10%-90%) improvement in symptoms.

#### Acceptability and Engagement

Treatment acceptability was similar in the immediate iDBT group (mean 36.7, SD 3.8) compared with the delayed iDBT group (mean 35.9, SD 5.3) at week 12; there was no difference between groups (t_56_=0.66; *P*=.51). A summary of the engagement metrics is provided in Table 3. On average, participants used the app for 2 hours and 24 minutes over the course of 43 days during the study, with 10 unique sign-in days. Participants also recorded an average of 543 meaningful interactions with the website. A breakdown of engagement by module is also provided in Table 3. All metrics tended to be higher on average in the immediate versus delayed iDBT group, consistent with the waitlist control design. Metrics improved further after removing 9 dropout participants who did not use the app after the first day. These individuals appeared to abide closer to the recommendation of using the resource for 8 days within the first month.

**Table 3:** Pocket Skills engagement metrics across groups.

			Started intervention (n=68), mean (SD; range)		Immediate iDBT^a^ (n=38), mean (SD; range)		Delayed iDBT (n=30), mean (SD; range)		Continued intervention after the first day (n=59), mean (SD; range)
**Overall engagement**									
	Total time	2 h 24 min (2 h 45 min; 0 min to 16 h 30 min)		2 h 49 min (3 h 6 min; 0 min to 16 h 30 min)		1 h 51 min (2 h 12 min; 0 min to 8 h 56 min)		2 h 45 min (2 h 47 min; 3 min to 16 h 30 min)	
	Interactions (clicks, page views, and inputs)	542.78 (569.46; 0-2830)		627.47 (576.22; 0-2468)		429.17 (560.28; 2-2830)		621.85 (571.36; 43-2830)	
	Unique days of log-in	10.24 (10.81; 1-64)		11.66 (12.68; 1-64)		8.45 (7.80; 1-36)		11.64 (10.94; 2-64)	
	Days spread	43.41 (32.65; 1-141)		50.95 (37.64; 1-141)		33.69 (22.42; 1-64)		49.88 (30.16; 2-141)	
	Days in the first 4 wk	6.69 (5.51; 1-29)		7.08 (5.88; 1-29)		6.17 (5.15; 1-21)		7.56 (5.41; 1-29)	
	Time in the first 4 wk	1 h 54 min (2 h 17 min; 0 min to 13 h 10 min)		2 h 9 min (2 h 30 min; 0 min to 13 h 10 min)		1 h 32 min (2 h 0 min; 1 min to 8 h 30 min)		2 h 10 min (2 h 20 min; 0 min to 13 h 10 min)	
	Web interactions in the first 4 wk	422.82 (474.87; 0-2566)		463.74 (455.99; 0-2143)		362.83 (507.54; 2-2566)		483.59 (481.66; 0-2566)	
**Module engagement**									
	General	38 min 19 s (1 h 21 min 35 s; 0 to 10 h 43 min)		48 min 50 s (1 h 43 min, 5 s; 0 to 10 h 36 min)		25 min 24 s (39 min 11 s; 0 min 52 s to 3 h 26 min)		43 min 38 s (1 h 26 min 25 s; 2 min 25 s to 3 h 26 min)	
	Mindfulness	46 min 17 s (41 min 43 s; 0 to 2 h 44 min)		53 min 46 s (43 min 35 s; 0 to 2 h 42 min)		36 min 58 s (38 min 29 s; 0 to 2 h 44 min)		53 min 21 s (40 min 21 s; 0 to 2 h 44 min)	
	Distress tolerance	11 min 0 s (20 min 38 s; 0 to 1 h 34 min)		13 min 06 s (20 min 54 s; 0 to 1 h 13 min)		8 min 34 s (20 min 40 s; 0 to 1 h 34 min)		12 min 41 s (21 min 41 s; 0 to 1 h 34 min)	
	Emotion regulation	32 min 38 s (46 min 56 s; 0 to 2 h 23 min)		36 min 31 s (51 min 31 s; 0 to 2 h 23 min)		28 min 39 s (41 min, 8 s; 0 to 2 h 10 min)		37 min 37 s (48 min 31 s; 0 to 2 h 23 min)	
	Interpersonal effectiveness	6 min 35 s (12 min 53 s; 0 to 48 min 39 s)		8 min 05 s (14 min 24 s; 0-48 min 39 s)		3 min 49 s (9 min 41 s; 0 to 34 min 33 s)		7 min 35 s (13 min 34 s; 0 to 48 min 39 s)	
	Addiction	9 min 18 s (19 min 3 s; 0 to 1 h 19 min)		8 min 52 s (17 min 42 s; 0 to 1 h 12 min)		8 min 19 s (19 min 31 s; 0 to 1 h 19 min)		10 min 43 s (20 min 6 s; 1 h to 19 min)	

^a^iDBT: internet-delivered dialectical behavioral therapy.

### Hypothesis 2: Were Improvements Greater in the Immediate Versus Delayed iDBT Group?

#### Overview

The unadjusted means, SDs, and the number of participants for each continuous variable are presented for each group and assessment point in Table 4, with between- and within-group effect size estimates presented in Table 5. The unadjusted values for each ordinal variable are provided in Multimedia Appendix 2.

**Table 4:** Unadjusted means (and SDs) by group and time point for continuous outcome measures.

Group and time				Continuous outcomes^a^							
			SDS^b^		PHQ-9^c^	GAD-7^d^	SBQ^e^	DERS-16^f^	MAAS^g^	WHODAS^h^	DBT-WCCL^i^
**Immediate iDBT^j^ (n=38)**											
	**Week 0**										
		Values, n (%)^k^	38 (100)		38 (100)	38 (100)	38 (100)	38 (100)	38 (100)	38 (100)	38 (100)
		Values, mean (SD)	8.7 (3.5)		12.9 (5.9)	11.9 (5.4)	8.6 (3.6)	51.8 (13.4)	3.6 (0.9)	16.5 (9.0)	1.8 (0.4)
	**Week 4**										
		Values, n (%)	34 (89)		34 (89)	34 (89)	34 (89)	33 (87)	31 (82)	31 (82)	31 (82)
		Values, mean (SD)	6.5 (4.3)		9.0 (4.9)	9.25 (4.5)	7.9 (3.3)	47.6 (13.5)	3.6 (0.8)	14.9 (8.6)	1.8 (0.5)
	**Week 8**										
		Values, n (%)	28 (74)		28 (74)	28 (74)	28 (74)	28 (74)	27 (71)	27 (71)	27 (71)
		Values, mean (SD)	6.3 (3.9)		9.5 (5.4)	8.7 (5.0)	7.8 (2.7)	44.1 (12.4)	4.0 (0.9)	12.4 (7.3)	1.9 (0.5)
	**Week 12**										
		Values, n (%)	30 (79)		30 (79)	30 (79)	30 (79)	30 (79)	29 (76)	29 (76)	29 (76)
		Values, mean (SD)	6.2 (4.1)		8.5 (4.6)	8.5 (4.3)	7.6 (3.1)	44.4 (10.8)	4.0 (0.8)	12.2 (6.9)	1.9 (0.5)
**Delayed iDBT (n=34)**											
	**Week 0**										
		Values, n (%)	34 (100)		34 (100)	34 (100)	34 (100)	34 (100)	34 (100)	34 (100)	34 (100)
		Values, mean (SD)	6.8 (3.5)		11.1 (6.7)	8.9 (6.1)	6.9 (3.5)	50.5 (12.1)	3.5 (1.0)	15.5 (9.2)	1.8 (0.4)
	**Week 4**										
		Values, n (%)	34 (100)		34 (100)	34 (100)	34 (100)	34 (100)	33 (97)	33 (97)	33 (97)
		Values, mean (SD)	5.6 (3.9)		10.1 (6.6)	8.6 (6.1)	6.9 (3.4)	49.9 (13.2)	3.7 (1.0)	14.3 (9.0)	1.7 (0.4)
	**Week 8**										
		Values, n (%)	28 (82)		28 (82)	28 (82)	28 (82)	28 (82)	28 (82)	28 (82)	28 (82)
		Values, mean (SD)	5.4 (3.9)		7.6 (6.1)	7.6 (5.4)	5.9 (3.3)	44.6 (13.5)	3.8 (0.9)	11.8 (9.3)	1.9 (0.5)
	**Week 12**										
		Values, n (%)	28 (82)		28 (82)	28 (82)	28 (82)	28 (82)	28 (82)	28 (82)	28 (82)
		Values, mean (SD)	5.3 (3.9)		7.3 (5.3)	7.1 (4.6)	6.1 (3.3)	43.1 (12.9)	4.1 (1.1)	11.1 (9.2)	2.0 (0.4)

^a^Descriptive statistics for variables with ordinal values (all secondary outcome variables) are presented in Multimedia Appendix 2.

^b^SDS: Substance Dependence Scale.

^c^PHQ-9: Patient Health Questionnaire-9.

^d^GAD-7: Generalized Anxiety Disorder-7.

^e^SBQ: Suicidal Behaviors Questionnaire.

^f^DERS-16: Difficulties in Emotion Regulation Scale-16 item.

^g^MAAS: Mindful Attention Awareness Scale.

^h^WHODAS: World Health Organization Disability Assessment Schedule.

^i^DBT-WCCL: Dialectical Behavior Therapy Ways of Coping Checklist.

^j^iDBT: internet-delivered dialectical behavioral therapy.

^k^n=number of participants contributing to the calculations.

**Table 5:** Effect sizes for continuous outcome measures.

Outcome	Within-group effect sizes, Cohen *d*^a^					Between-group effect sizes, Cohen *d*^b^	
	Immediate iDBT^c^		Delayed iDBT		Immediate iDBT vs delayed iDBT		
	Week 4	Week 12	Week 4^d^	Week 12^e^	Week 4 vs baseline		Week 12 vs baseline
SDS^f^	−0.58	−0.95	−0.73	−0.84	−0.32		−0.32
PHQ-9^g^	−0.66	−0.87	−0.42	−1.05	−0.48		−0.04
GAD-7^h^	−1.70	−1.52	−0.27	−1.07	−0.40		−0.21
SBQ^i^	−0.27	−0.56	0.02	−0.45	−0.18		−0.01
DERS-16^j^	−0.58	−0.68	−0.11	−1.23	−0.27		0.01
MAAS^k^	−0.02	3.78	0.78	2.34	−0.22		−0.21
WHODAS^l^	−0.28	−0.76	−0.51	−3.42	−0.04		0.03
DBT-WCCL^m^	0.16	0.25	−0.38	0.48	0.22		−0.22

^a^We calculated Cohen repeated measures *d*, with a pooled SD (refer to the study by Lakens [61], formula 8) with values ≥0.20=small, ≥0.50=medium or moderate, and ≥0.80=large effect [62].

^b^Uses the Klauer method, where effect size, Cohen *d* for both groups was calculated and then subtracted from each other. This allowed for the correction of different sample sizes and baseline values.

^c^iDBT: internet-delivered dialectical behavioral therapy.

^d^This effect size technically captures the repeated baseline effect.

^e^This effect size technically captures 8 weeks following the start of the intervention.

^f^SDS: Substance Dependence Scale.

^g^PHQ-9: Patient Health Questionnaire-9.

^h^GAD-7: Generalized Anxiety Disorder-7.

^i^SBQ: Suicidal Behaviors Questionnaire.

^j^DERS-16: Difficulties in Emotion Regulation Scale-16 item.

^k^MAAS: Mindful Attention Awareness Scale.

^l^WHODAS: World Health Organization Disability Assessment Schedule.

^m^DBT-WCCL: Dialectical Behavior Therapy Ways of Coping Checklist.

#### Primary Outcome

Contrary to our hypotheses, we did not find any significant group×time interactions for the severity of substance dependence at week 4 or week 12.

#### Secondary Outcomes

Consistent with the hypotheses, the results supported greater benefits for the immediate versus delayed iDBT group for several secondary outcomes. At week 4, there were significant group×time interactions for depression and anxiety, where the immediate access group reported fewer depressive (b=−2.46; SE 1.05; 95% CI −4.51 to −0.40; *P=*.02) and anxiety symptoms (b=−2.22; SE 0.96; 95% CI −4.09 to −0.34; *P=*.02) compared with the delayed iDBT group. At week 12, there were significant group×time interactions for standard alcoholic drinks per day (b*=*−2.00; SE 0.83; 95% CI −3.64 to −0.36; *P=*.02) and nonalcoholic substance use (b*=*−3.74; SE 1.47; 95% CI −6.63 to −0.85; *P=*.01), where the immediate group had lower frequencies of both over the course of the study compared with the delayed group. Contrary to the expectations, there were no significant group×time interactions for all other outcomes: emotion dysregulation, suicidality, DBT skills acquisition, dispositional mindfulness, functional disability, and risky impulsive behaviors.

### Hypothesis 3: Did iDBT Produce Improvements Regardless of Group?

#### Primary Outcome

There were significant main effects of time at week 4 (b*=*−1.73; SE 0.34; 95% CI −2.40 to −1.07; *P*<.001) and week 12 (b=−2.09; SE 0.36; 95% CI −2.80 to −1.39; *P*<.001), indicating a significant decrease in substance dependence for both groups, with no differences between groups (*P=*.25).

#### Secondary Outcomes

There were several findings supporting the benefits of the intervention in the follow-up phase of the study (no other significant main effects emerged at week 4). At week 12, there were significant main effects of time for depression (b=−2.95; SE 0.79; 95% CI −4.50 to −1.39; *P*<.001), anxiety (b=−1.57; SE 0.73; 95% CI −2.99 to −0.14; *P*=.03), suicidality (b=−0.70; SE 0.24; 95% CI −1.12 to −0.28; *P*=.001), emotion dysregulation (b=−6.56; SE 1.20; 95% CI −8.90 to −4.21; *P*<.001), functional disability (b=−3.64; SE 0.77; 95% CI −5.15 to −2.14; *P*<.001), dispositional mindfulness (b=0.44; SE 0.09; 95% CI 0.27-0.62; *P*<.001), and DBT skill acquisition (b=0.14; SE=0.05; 95% CI 0.04-0.23; *P*=.005), indicating that both groups saw significant improvements from baseline over the study duration, with no differences between groups (all *P*>.25). There were no main effects of time for risky impulsive behaviors and no difference between groups (*P*>.23).

## Discussion

### Summary

This study is unique in that it delivered high-quality iDBT in a self-guided format that participants could use through any internet browser on a computer, tablet, or smartphone. Here, we evaluated the feasibility, acceptability, and potential efficacy of iDBT in a sample of treatment-seeking individuals with SUDs often presenting with additional mental health symptoms. In this study, *Pocket Skills* 2.0 garnered some meaningful support as a potential intervention for those with SUDs and other mental health concerns. We also discuss some caveats and limitations in the following sections.

### Feasibility and Acceptability

The intervention was deemed credible and potentially helpful by participants. In terms of treatment initiation, we found that 94% of the randomized participants started *Pocket Skills* compared with 98% in a previous remote iDBT intervention study [30]. For reference, 88% of the participants started the intervention in the study by van Spijker et al [28] and only 39% of the participants started self-guided iDBT in the study by Simon et al [31]. In this study, not initiating iDBT was mostly because of participants not attending a follow-up session after 4 weeks of being on the waitlist, and in 1 case, owing to technical issues. Thus, the feasibility of deploying iDBT remains high with few technical compatibility issues. These results support the feasibility of adapting DBT for delivery in internet-delivered formats in this context [25,30,34].

Of those who started iDBT, 13% (9/68) were early dropouts, defined as those who did not attempt the intervention after the first day of use, and 50% (34/68) continued to use the app after 4 weeks. Comparatively, Wilks et al [30] recorded a dropout rate of 19%, although different dropout criteria were used (eg, stopped attempting or completing the intervention for 3 weeks in a row). This study differs in that our participants had unrestricted access to all content, whereas the previous study used a week-to-week module approach; thus, we had different definitions of dropout by virtue of study design. There was an overall dropout rate of approximately 10% in the intervention arm in the study by van Spijker et al [28], whereas <9% went beyond the introduction section in the study by Simon et al [31,32]. The rate can also be compared with internet-based psychological treatments more broadly (31%), in-person delivered DBT for different clinical conditions (28%), and in-person psychological treatments for SUDs (30%) [63-65]. Although definitions of dropout vary across trials, this study provides some promise regarding the potential uptake and adherence to iDBT by individuals with SUDs. An analysis of the predictors of dropout from the 2018 study [30] indicated that technological barriers and low perceptions of usefulness emerged as significant [33]. Although we focused on the current hypotheses, we intend to examine predictors of treatment outcomes (including dropout) in future research.

The time spent on the iDBT intervention varied widely. Only 35% (24/68) of the participants completed the recommended dose of spending 8 days in the first 4 weeks, which improved to 41% (24/59) when early dropouts were omitted. These findings can be contextualized by the limited support and self-guided nature of the intervention. Comparatively, 42% of the participants in the study by Wilks et al [30] completed half of the iDBT content in the same time frame (1 month), which included considerably more support, such as daily reminders, homework assignments, and phone calls regarding suicide risk. Approximately half of the participants in the intervention arm completed ≥3 of 6 sessions in the study by van Spijker et al [28], and a similar proportion finished a 15-session course of DBT delivered by email [27]. While we did offer text message reminders to facilitate encouragement, few participants asked for additional meetings or followed our suggested guide. However, even with limited support, a sizeable proportion of participants used the app over several days within the first month, totaling >1 hour of use. These findings suggest potential benefits of additional meetings or coaching sessions, which may improve adherence and engagement to the intervention and improve clinical outcomes overall.

There were relatively high ratings for the content, suitability, and trustworthiness of *Pocket Skills* using an established measure of treatment acceptability, extending 2 earlier studies [30,34]. Participants recorded most of their engagement within the first 4 weeks of the intervention and in that time, averaged 2 hours of interaction. In a previous 4-week trial of an earlier version of *Pocket Skills*, which was conducted in patients concurrently completing in-person DBT, participants used the app for 14 out of 28 days and spent 2.25 hours on the app during that time [34]. This equated to approximately 4 minutes of activity per person per day. Given that our study was largely self-guided without additional support or check-ins, this newer version of *Pocket Skills* saw comparative engagement with respect to total time. One self-guided skills training intervention reported 10.5 hours of use on average or approximately 15 minutes per day over 6 weeks [28]. Examining iDBT as an adjunct to in-person (or videoconference) DBT to improve engagement even further may be warranted.

### Potential Efficacy

With regard to our second hypothesis evaluating the waitlist control design of the study, we found little evidence that the immediate iDBT group benefited more in the acute period of 4 weeks. We did not find a difference between groups in our primary outcome at this time point, where we expected it. We found interactions at week 4 for 2 secondary outcomes (ie, depression and anxiety) in favor of the hypothesis. We found additional interactions during the follow-up period (week 12) for decreased standard alcoholic drinks per day and overall substance use frequency in favor of the immediate iDBT group. Notably, we originally planned a 16-week trial with an equal immediate and waitlist period of 8 weeks. However, in a pilot study, we found attrition and lack of engagement to be greater than in this study, which contributed to the revised study design. Most studies on app- and internet-based interventions use one month as an acute test of the intervention with another month as a follow-up assessment period owing to increasing attrition after 7 to 8 weeks [35-37].

Our third hypothesis regarding overall improvement was more consistently supported, with favorable improvements in our primary and secondary outcome measures in both groups by week 12. There were medium to large effect size improvements in our primary outcome of substance dependence, both in the acute (week 4) and follow-up phase, consistent with the literature supporting in-person DBT for alcohol use disorders and SUDs [16,21-24]. There were many medium to large effect size improvements to our secondary outcomes by week 12, including depression, anxiety, suicidal behavior, emotion dysregulation, and functional disability and these effects did not differ significantly by group once accounting for baseline differences. These findings are consistent with in-person DBT improvements in suicidal behavior, depression, anxiety, and emotion dysregulation [23,66-69] and extend a previous stand-alone iDBT study [30]. The intervention also improved DBT skill acquisition and dispositional mindfulness in both groups by week 12, as seen by small-to-large positive effect size values and in line with the literature findings on face-to-face DBT [52,70,71]. Whether DBT skill acquisition mediated treatment outcomes in this study, as implied by previous research [68,69], is a hypothesis that could be examined in a follow-up analysis.

### Limitations

Our ability to detect interactions during the acute phase of treatment was limited given that differences would have had to be medium or large to detect using our unbalanced waitlist design. The use of a waitlist control may have led some individuals to consider other treatment options or drop out of the study without connecting to the intervention, especially given its unblinded nature following randomization. Alternatively, the waitlist condition may have supported a nocebo effect as participants reported favorable changes in most outcomes despite a lack of treatment [72]. Although the randomization procedure was conducted in a blind manner, following the baseline session, all other follow-ups and contact with participants were unblinded, which may have introduced potential experimenter bias. The primary and most secondary outcomes were participant rated (ie, self-report), which is also less robust to bias than a blinded outcomes assessor.

In terms of feasibility metrics, although we saw that roughly half of the participants remained active on iDBT after 4 weeks, it is not clear how consistently active they were. Because we did not restrict access to other interventions, it remains unclear how specific the intervention effects were tied to iDBT in this study. We attempted to mitigate concerns about this by asking questions about different services that participants were receiving at each follow-up and found little to no increase in psychotropic medications, community support groups, and additional treatments. More analyses are needed to understand the dose-response relationship between the intervention and treatment outcome, which will be addressed in future work.

*Pocket Skills* 2.0 omits several aspects of in-person formats of DBT that may improve engagement and adherence, such as a significant group therapy component; more robust tracking of thoughts, emotions, and behaviors using diary cards; and handouts and worksheets for homework. These implementation differences compared with standard in-person DBT may have influenced treatment outcomes. As a technical limitation, most participants accessed iDBT on a home computer or laptop. Although we discussed the ability to sign in on mobile devices with participants, we typically asked them to sign in for the first time on a computer or laptop based on experiences during the piloting phase of this study (ie, there was more difficulty signing in on the mobile iOS platform). This procedural issue may have introduced a barrier to using iDBT on mobile devices; however, we could have spent more time ensuring that the intervention was working on both participants’ smartphones and computer devices. Future implementations could include subsequent meetings with participants (eg, check-ins) to address any technological and compatibility issues more quickly.

Despite efforts to recruit a diverse sample, our sample was predominantly White, female, heterosexual, and largely aged between 18 and 45 years. Future research should attempt to replicate the outcomes of iDBT across more diverse samples, such as sexual, gender, and ethnoracial minority groups (refer to the study by Harned et al [73] for review). Our sample was heterogeneous with respect to their endorsement of alcohol or nonalcoholic substance difficulties. Measuring the frequency and severity of multiple substances in an efficient way is challenging. Owing to the use of different measures and rating scales to assess alcohol and nonalcoholic substances (as well as risky and impulsive behaviors), our raw data required rescaling to create new ordinal scales that approximately modeled the same frequency and severity across multiple scales. Future research could adhere to more standardized approaches that allow for responses to be collected and then coded more reliability. For example, the Timeline Follow Back interview has been used to self-report alcohol and substance use as well as risky sexual behaviors [74,75].

### Conclusions

Notwithstanding the limitations of this study, our iDBT intervention *Pocket Skills* 2.0 was supported as a feasible and acceptable intervention for those with SUDs and other mental health concerns. However, methods to improve engagement should be further evaluated. The intervention not only showed potential effectiveness for substance dependence but also demonstrated positive effects across various mental health symptoms, affirming its clinical utility. These findings add to the sparse literature on internet-based DBT and internet-delivered psychological interventions for SUDs. This format has the potential to increase accessibility and reduce the costs and resources required for in-person DBT. Several research priorities were identified to potentially improve engagement and optimize treatment outcomes as well as understand how our iDBT intervention can be integrated into the larger landscape of treatment options for SUDs and other conditions.

## Appendix

Multimedia Appendix 1 CONSORT-eHEALTH checklist (V 1.6.1).

Multimedia Appendix 2 Supplementary results and tables.

## Abbreviations

CONSORT: Consolidated Standards of Reporting Trials
CONSORT-EHEALTH: Consolidated Standards of Reporting Trials of Electronic and Mobile Health Applications and Online Telehealth
DBT: dialectical behavior therapy
iDBT: internet-delivered dialectical behavior therapy
RCT: randomized controlled trial
REDCap: Research Electronic Data Capture
SUD: substance use disorder

## Data Availability

Deidentified data may be requested from ARD.
