# Supplementary material for: Feasibility, Acceptability, and Potential Efficacy of a Self-Guided Internet-Delivered Dialectical Behavior Therapy Intervention for Substance Use Disorders: Randomized Controlled Trial
Source: JMIR Ment Health. 2024 Jan 16;11:e50399. doi: 10.2196/50399 (PMC10828941; doi:10.2196/50399)
Supplement: Multimedia Appendix 2 [file mental_v11i1e50399_app2.docx]

**Supplementary Materials**

**Table S1: Comparison of the current iDBT intervention compared to previous research.**

|  | **Wilks et al. (2018)** | **Schroeder et al. (2018)** | **Current Evaluation** |
| --- | --- | --- | --- |
| **Version of Pocket Skills** | Pre-dates Pocket Skills | Version 1.0 | Version 2.0 |
| **Content** | Mindfulness  Addiction  Emotion Regulation  Distress Tolerance | Mindfulness*  Addiction*  Emotion Regulation*  Distress Tolerance* | Mindfulness*  Distress Tolerance*  Emotion Regulation*  Interpersonal Effectiveness**  Addiction* |
| **Therapist Support** | Therapist-guided with weekly homework sent via email, reminders, and support calls | Adjunct to therapist led face-to-face DBT. | Limited therapist support (e.g., therapist provided introduction and demo of iDBT only). |
| **Length of Intervention** | 8 weeks | Not reported. | 4 weeks |
| **Design of Study** | Waitlist-controlled RCT | Single-arm observational study | Waitlist-controlled RCT |
| **Length of Study** | 16 weeks | 4 weeks | 12 weeks |
| **Post-Baseline Assessments** | 4, 8, 12, and 16 weeks | 1, 2, 3, and 4 weeks | 4, 8, and 12 weeks |
| **Incentives for Participation** | $120 maximum | $200 maximum | $70 maximum |
| **Target Sample** | Heavy drinkers with suicidal ideation (based on thresholds on a scale). | Adults participating in DBT, with predominant presentations of depression generalized anxiety disorder, and borderline personality disorder | People with a current alcohol or substant use disorder (SUDs), with comorbid conditions allowed. |
| **Recruitment Source** | Online web advertisements | Online DBT listserv | Hospital and community setting |
| **Technological Format** | Web-based electronic learning server (Articulate Online) only accessible on a computer. Skills lessons were delivered via presentations with videos and graphics. | Website portal on Microsoft Azure accessible on any internet browser. Skills lessons were delivered via embedded videos and a chatbot. | Website portal on Microsoft Azure accessible on any internet browser, but with an emphasis on computers and laptops. Skills lessons were delivered via embedded videos and a chatbot. |
| **Format of Delivery** | Sequential (finish one module before moving to next) | Semi-Sequential (starting content in one module opens next module) | All modules freely available from the start of the intervention. |

Note: *Content was revised compared to previous iteration. **Content was introduced for the first time.

**Supplementary Results**

We report here any significant Group × Time interactions or main effects of Time for Week 8. There was a Group × Time interaction for anxiety at Week 8, *b* = -2.62, *SE* = 1.01, *p* = .01. There were main effects for Time at Week 8 for substance dependence (*b* = -1.92, *SE* = .36, *p* < .0001), depression (*b* = -2.14, *SE* = .79, *p* = .008), suicidality (*b* = -.63, *SE* = .21, *p* = .003), emotion dysregulation (*b* = -5.76, *SE* = 1.20, *p* < .0001), functional disability (*b* = -3.19, *SE* = .77, *p* < .0001), dispositional mindfulness (*b* = .30, *SE* = .09, *p* = .001) and risky sexual behaviors (*b* = -2.11, *SE* = .94, *p* = .03). There was no significant main effect for Time at Week 8 for DBT skills. For confidence intervals, please see below output tables.

The following are supplementary tables represent the final model output for each dependent variable. We provide these for full transparency given the number of variables included in our clinical trial registration. As a reminder, each dependent variable was assessed with models containing an interaction effect (Group × Time, as factor variables) and main effects only (Group + Time, as factor variables) along with a continuous covariate controlling for the baseline assessment of each outcome per person. The final model chosen for interpretation was the better fitting model based on lower AIC and BIC values. Thus, if the model fit was improved by the inclusion of the interaction term, we report that model; if not, we removed the interaction and report the model with the main effects only.

**Table S2. Substance Dependence**

|  | **SDS total** | | | | |
| --- | --- | --- | --- | --- | --- |
| *Predictors* | *Estimates* | *SE* | *95% CI* | *t* | *p* |
| (Intercept) | 8.06 | 0.36 | 7.36, 8.77 | 22.42 | **<0.001** |
| Week 4 vs. Baseline | -1.73 | 0.34 | -2.40, -1.07 | -5.10 | **<0.001** |
| Week 8 vs. Baseline | -1.92 | 0.36 | -2.62, -1.22 | -5.38 | **<0.001** |
| Week 12 vs. Baseline | -2.09 | 0.36 | -2.80, -1.39 | -5.84 | **<0.001** |
| Group (1 vs. 2) | -0.49 | 0.42 | -1.31, 0.33 | -1.16 | 0.247 |
| SDS Baseline Covariate | 0.88 | 0.06 | 0.77, 1.00 | 14.94 | **<0.001** |
|  | **Random Effects** | | | | |
| σ^2^ | 4.05 | | | |  |
| τ_00_ _PID_ | 1.74 | | | |  |
| ICC | 0.30 | | | |  |
| N _PID_ | 72 | | | |  |
| Observations | 261 | | | |  |
| Marginal R^2^ / Conditional R^2^ | 0.636 / 0.746 | | | |  |

**Table S3. Depression**

|  | **PHQ-9 total** | | | | |
| --- | --- | --- | --- | --- | --- |
| *Predictors* | *Estimates* | *SE* | *95% CI* | *t* | *p* |
| (Intercept) | 11.41 | 0.63 | 10.17, 12.64 | 18.10 | **<0.001** |
| Week 4 vs. Baseline | -1.06 | 0.75 | -2.53, 0.41 | -1.41 | 0.161 |
| Week 8 vs. Baseline | -2.14 | 0.79 | -3.69, -0.58 | -2.69 | **0.008** |
| Week 12 vs. Baseline | -2.95 | 0.79 | -4.50, -1.39 | -3.71 | **<0.001** |
| Group (1 vs. 2) | 0.47 | 0.87 | -1.24, 2.18 | 0.54 | 0.591 |
| PHQ-9 Baseline Covariate | 0.73 | 0.05 | 0.63, 0.83 | 14.69 | **<0.001** |
| Week 4 vs. Baseline × Group (1 vs. 2) | -2.46 | 1.05 | -4.51, -0.40 | -2.35 | **0.020** |
| Week 8 vs. Baseline × Group (1 vs. 2) | -0.48 | 1.11 | -2.66, 1.69 | -0.44 | 0.662 |
| Week 12 vs. Baseline × Group (1 vs. 2) | -0.64 | 1.10 | -2.80, 1.52 | -0.58 | 0.563 |
|  | **Random Effects** | | | | |
| σ^2^ | 9.64 | | | |  |
| τ_00_ _PID_ | 3.86 | | | |  |
| ICC | 0.29 | | | |  |
| N _PID_ | 72 | | | |  |
| Observations | 260 | | | |  |
| Marginal R^2^ / Conditional R^2^ | 0.627 / 0.734 | | | |  |

**Table S4. Anxiety**

| **GAD-7 total** | | | | | |
| --- | --- | --- | --- | --- | --- |
| *Predictors* | *Estimates* | *SE* | *95% CI* | *t* | *p* |
| (Intercept) | 9.93 | 0.58 | 8.79, 11.08 | 17.05 | **<0.001** |
| Week 4 vs. Baseline | -0.32 | 0.69 | -1.67, 1.02 | -0.47 | 0.639 |
| Week 8 vs. Baseline | -0.54 | 0.73 | -1.96, 0.88 | -0.75 | 0.456 |
| Week 12 vs. Baseline | -1.57 | 0.73 | -2.99, -0.14 | -2.16 | **0.032** |
| Group (1 vs. 2) | 0.90 | 0.81 | -0.69, 2.49 | 1.11 | 0.267 |
| GAD-7 Baseline Covariate | 0.71 | 0.05 | 0.61, 0.80 | 14.36 | **<0.001** |
| Week 4 vs. Baseline × Group (1 vs. 2) | -2.22 | 0.96 | -4.09, -0.34 | -2.32 | **0.021** |
| Week 8 vs. Baseline × Group (1 vs. 2) | -2.62 | 1.01 | -4.61, -0.64 | -2.59 | **0.010** |
| Week 12 vs. Baseline × Group (1 vs. 2) | -1.41 | 1.01 | -3.39, 0.57 | -1.40 | 0.164 |
| **Random Effects** | | | | | |
| σ^2^ | 8.05 | | | | |
| τ_00_ _PID_ | 3.32 | | | | |
| ICC | 0.29 | | | | |
| N _PID_ | 72 | | | | |
| Observations | 260 | | | | |
| Marginal R^2^ / Conditional R^2^ | 0.618 / 0.729 | | | | |

**Table S5. Suicidality**

| **SBQ-4 total** | | | | | |
| --- | --- | --- | --- | --- | --- |
| *Predictors* | *Estimates* | *SE* | *95% CI* | *t* | *p* |
| (Intercept) | 7.73 | 0.21 | 7.32, 8.13 | 37.46 | **<0.001** |
| Week 4 vs. Baseline | -0.26 | 0.20 | -0.66, 0.13 | -1.27 | 0.205 |
| Week 8 vs. Baseline | -0.63 | 0.21 | -1.05, -0.21 | -2.96 | **0.003** |
| Week 12 vs. Baseline | -0.70 | 0.21 | -1.12, -0.28 | -3.28 | **0.001** |
| Group (1 vs. 2) | -0.04 | 0.24 | -0.50, 0.42 | -0.16 | 0.875 |
| SBQ4 Baseline Covariate | 0.84 | 0.03 | 0.78, 0.91 | 25.81 | **<0.001** |
| **Random Effects** | | | | | |
| σ^2^ | 1.45 | | | | |
| τ_00_ _PID_ | 0.52 | | | | |
| ICC | 0.26 | | | | |
| N _PID_ | 72 | | | | |
| Observations | 260 | | | | |
| Marginal R^2^ / Conditional R^2^ | 0.825 / 0.871 | | | | |

**Table S6. Emotion dysregulation**

| **DERS total** | | | | | |
| --- | --- | --- | --- | --- | --- |
| *Predictors* | *Estimates* | *SE* | *95% CI* | *t* | *p* |
| (Intercept) | 51.67 | 1.15 | 49.42, 53.93 | 44.89 | **<0.001** |
| Week 4 vs. Baseline | -2.12 | 1.15 | -4.37, 0.13 | -1.84 | 0.066 |
| Week 8 vs. Baseline | -5.76 | 1.20 | -8.12, -3.41 | -4.79 | **<0.001** |
| Week 12 vs. Baseline | -6.56 | 1.20 | -8.90, -4.21 | -5.47 | **<0.001** |
| Group (1 vs. 2) | -1.16 | 1.29 | -3.69, 1.37 | -0.90 | 0.370 |
| DERS Baseline Covariate | 0.78 | 0.05 | 0.68, 0.88 | 15.18 | **<0.001** |
| **Random Effects** | | | | | |
| σ^2^ | 45.49 | | | | |
| τ_00_ _PID_ | 16.50 | | | | |
| ICC | 0.27 | | | | |
| N _PID_ | 72 | | | | |
| Observations | 258 | | | | |
| Marginal R^2^ / Conditional R^2^ | 0.628 / 0.727 | | | | |

**Table S7. Dispositional Mindfulness**

| **MAAS Average Score** | | | | | |
| --- | --- | --- | --- | --- | --- |
| *Predictors* | *Estimates* | *SE* | *95% CI* | *t* | *p* |
| (Intercept) | 3.59 | 0.09 | 3.42, 3.77 | 40.57 | **<0.001** |
| Week 4 vs. Baseline | 0.12 | 0.09 | -0.05, 0.29 | 1.42 | 0.16 |
| okWeek 8 vs. Baseline | 0.30 | 0.09 | 0.12, 0.48 | 3.32 | **0.001** |
| Week 12 vs. Baseline | 0.44 | 0.09 | 0.27, 0.62 | 4.93 | **<0.001** |
| Group (1 vs. 2) | -0.05 | 0.10 | -0.25, 0.15 | 0.49 | 0.627 |
| MAAS Baseline Covariate | 0.76 | 0.05 | 0.65, 0.86 | 14.22 | **<0.001** |
| **Random Effects** | | | | | |
| σ^2^ | 0.25 | | | | |
| τ_00_ _PID_ | 0.11 | | | | |
| ICC | 0.30 | | | | |
| N _PID_ | 72 | | | | |
| Observations | 254 | | | | |
| Marginal R^2^ / Conditional R^2^ | 0.611 / 0.727 | | | | |

**Table S8. Functional disability**

| **WHODAS 2.0 total** | | | | | |
| --- | --- | --- | --- | --- | --- |
| *Predictors* | *Estimates* | *SE* | *95% CI* | *t* | *p* |
| (Intercept) | 15.79 | 0.67 | 14.47, 17.10 | 23.52 | **<0.001** |
| Week 4 vs. Baseline | -1.46 | 0.74 | -2.91, -0.01 | -1.98 | 0.05 |
| Week 8 vs. Baseline | -3.19 | 0.77 | -4.70, -1.68 | -4.15 | **<0.001** |
| Week 12 vs. Baseline | -3.64 | 0.77 | -5.15, -2.14 | -4.75 | **<0.001** |
| Group (1 vs. 2) | 0.06 | 0.71 | -1.34, 1.46 | 0.09 | 0.929 |
| WHODAS Baseline Covariate | 0.79 | 0.04 | 0.71, 0.86 | 19.95 | **<0.001** |
| **Random Effects** | | | | | |
| σ^2^ | 18.59 | | | | |
| τ_00_ _PID_ | 3.64 | | | | |
| ICC | 0.16 | | | | |
| N _PID_ | 72 | | | | |
| Observations | 254 | | | | |
| Marginal R^2^ / Conditional R^2^ | 0.709 / 0.757 | | | | |

**Table S9. DBT Skills Acquisition**

| **DBT WCCL (average item score)** | | | | | |
| --- | --- | --- | --- | --- | --- |
| *Predictors* | *Estimates* | *SE* | *95% CI* | *t* | *p* |
| (Intercept) | 1.79 | 0.04 | 1.70, 1.87 | 40.43 | **<0.001** |
| Week 4 vs. Baseline | -0.02 | 0.05 | -0.11, 0.07 | -0.37 | 0.711 |
| Week 8 vs. Baseline | 0.09 | 0.05 | -0.00, 0.19 | 1.91 | 0.057 |
| Week 12 vs. Baseline | 0.14 | 0.05 | 0.04, 0.23 | 2.83 | **0.005** |
| Group (1 vs. 2) | -0.00 | 0.05 | -0.10, 0.09 | -0.06 | 0.954 |
| DBT WCCL Baseline Covariate | 0.76 | 0.06 | 0.64, 0.88 | 12.65 | **<0.001** |
| **Random Effects** | | | | | |
| σ^2^ | 0.07 | | | | |
| τ_00_ _PID_ | 0.02 | | | | |
| ICC | 0.23 | | | | |
| N _PID_ | 72 | | | | |
| Observations | 254 | | | | |
| Marginal R^2^ / Conditional R^2^ | 0.519 / 0.628 | | | | |

**Table S10. Standard Alcoholic Drinks per day (Ordinal Values with increasing severity)**

| **DDQ: Standard Alcoholic Drinks per day  (average of ordinal value across 7 days of the week, rounded to nearest one)** | | | | |  |
| --- | --- | --- | --- | --- | --- |
| *Predictors* | *Estimates* | *SE* | *95% CI* | *Z* | *p* |
| Week 4 vs. Baseline | -0.24 | 0.49 | -1.21, 0.73 | -0.49 | 0.627 |
| Week 8 vs. Baseline | -0.63 | 0.53 | -1.68, 0.42 | -1.18 | 0.239 |
| Week 12 vs. Baseline | -0.19 | 0.52 | -1.21, 0.83 | -0.37 | 0.712 |
| Group (1 vs. 2) | -0.17 | 0.53 | -1.21, 0.86 | -0.33 | 0.744 |
| Standard Alcoholic Drinks Baseline Covariate | 2.28 | 0.25 | 1.79, 2.77 | 9.18 | **<0.001** |
| Week 4 vs. Baseline × Group (1 vs. 2) | -1.17 | 0.75 | -2.65, 0.30 | -1.56 | 0.118 |
| Week 8 vs. Baseline × Group (1 vs. 2) | -1.44 | 0.82 | -3.04, 0.16 | -1.76 | 0.079 |
| Week 12 vs. Baseline × Group (1 vs. 2) | -2.00 | 0.83 | -3.64, -0.36 | -2.40 | **0.017** |
| **Random Effects** | | | |  |  |
| τ_00_ _PID_ | 0.93 | | |  |  |
| N _PID_ | 72 | | |  |  |
| Observations | 260 | | |  |  |
| Log-likelihood | -209.98 | | |  |  |
| AIC | 447.95 | | |  |  |

**Table S11. Non-alcoholic Substance Use (Ordinal Values with increasing severity)**

| **Modified NIDA-ASSIST: Non-Alcoholic Substance Use in past month**  **(average of ordinal value across 8 substance categories, rounded to nearest one)** | | | | | |
| --- | --- | --- | --- | --- | --- |
| *Predictors* | *Estimates* | *SE* | *95% CI* | *Z* | *p* |
| Week 4 vs. Baseline | 0.0001 | 1.00 | -1.96, 1.96 | 0.0001 | > .999 |
| Week 8 vs. Baseline | 0.42 | 1.07 | -1.67, 2.52 | 0.40 | 0.692 |
| Week 12 vs. Baseline | 1.71 | 1.09 | -0.43, 3.84 | 1.56 | 0.118 |
| Group (1 vs. 2) | 0.87 | 1.17 | -1.42, 3.17 | 0.75 | 0.456 |
| Substance Use Baseline Covariate | 8.33 | 1.98 | 4.44, 12.21 | 4.20 | **<0.001** |
| Week 4 vs. Baseline × Group (1 vs. 2) | 0.19 | 1.28 | -2.31, 2.70 | 0.15 | 0.880 |
| Week 8 vs. Baseline × Group (1 vs. 2) | -1.77 | 1.39 | -4.49, 0.95 | -1.28 | 0.202 |
| Week 12 vs. Baseline × Group (1 vs. 2) | -3.74 | 1.47 | -6.63, -0.85 | -2.54 | **0.011** |
| τ_00_ _PID_ | 8.69 | | | | |
| N _PID_ | 72 | | | | |
| Observations | 260 | | | | |
| Log-likelihood | -95.98 | | | | |
| AIC | 215.96 | | | | |

**Table S12. Risky impulsive behaviors (Ordinal Values with increasing severity)**

| **RISQ: Risky Impulsive Behaviors**  **(average of ordinal value across 8 items, rounded to nearest one)** | | | | | |
| --- | --- | --- | --- | --- | --- |
| *Predictors* | *Estimates* | *SE* | *95% CI* | *Z* | *p* |
| Week 4 vs. Baseline | 0.48 | 0.40 | -0.30, 1.27 | 1.20 | 0.230 |
| Week 8 vs. Baseline | -0.31 | 0.44 | -1.19, 0.56 | -0.70 | 0.483 |
| Week 12 vs. Baseline | 0.35 | 0.42 | -0.46, 1.17 | 0.85 | 0.394 |
| Group (1 vs. 2) | 0.26 | 0.34 | -0.41, 0.94 | 0.77 | 0.443 |
| RISQ Baseline Covariate | 2.42 | 0.33 | 1.77, 3.07 | 7.33 | **<0.001** |
| τ_00_ _PID_ | 0.27 | | | | |
| N _PID_ | 72 | | | | |
| Observations | 255 | | | | |
| Log-likelihood | -163.58 | | | | |
| AIC | 347.15 | | | | |

**Table S13. Unadjusted Means (and Standard Deviations) by Group and Time Point for Ordinal Outcome Measures**

|  | Arm 1: Immediate Access | | | | | | | | Arm 2: Delayed Access | | | | | | | |
| --- | --- | --- | --- | --- | --- | --- | --- | --- | --- | --- | --- | --- | --- | --- | --- | --- |
|  | Week 0 | | Week 4 | | Week 8 | | Week 12 | | Week 0 | | Week 4 | | Week 8 | | Week 12 | |
| Measure | N | M(SD) | N | M(SD) | N | M(SD) | N | M(SD) | N | M(SD) | N | M(SD) | N | M(SD) | N | M(SD) |
| All Substances | 38 | 0.8 (0.7) | 34 | 0.7 (0.7) | 28 | 0.6 (0.7) | 30 | 0.6 (0.7) | 34 | 0.5 (0.6) | 34 | 0.5 (0.6) | 28 | 0.5 (0.6) | 28 | 0.6 (0.6) |
| Tobacco/Cannabis | 38 | 2.7 (2.0) | 34 | 2.6 (1.8) | 28 | 2.4 (2.1) | 30 | 2.1 (1.9) | 34 | 1.9 (1.7) | 34 | 1.7 (1.8) | 28 | 1.8 (1.8) | 28 | 1.8 (1.7) |
| Non-Tob/Cannabis | 38 | 0.2 (0.6) | 34 | 0.3 (0.4) | 28 | 0.3 (0.5) | 30 | 0.2 (0.5) | 34 | 0.1 (0.3) | 34 | 0.1 (0.4) | 28 | 0.1 (0.3) | 28 | 0.1 (0.3) |
| Standard Alcohol Drinks Per day | 38 | 1.0 (1.2) | 34 | 0.5 (0.8) | 28 | 0.4 (0.6) | 30 | 0.4 (0.8) | 34 | 1.1 (1.2) | 34 | 1.0 (1.2) | 28 | 0.9 (0.9) | 28 | 0.9 (1.0) |
| Risky Imp Behaviours | 38 | 1.6 (0.7) | 32 | 1.5 (0.7) | 27 | 1.5 (0.6) | 29 | 1.6 (0.9) | 34 | 1.2 (0.4) | 33 | 1.5 (0.6) | 28 | 1.2 (0.4) | 28 | 1.3 (0.5) |
| Risky Sexual Behaviours | 38 | 1.3 (0.7) | 32 | 1.1 (0.4) | 27 | 1.1 (0.6) | 29 | 1.2 (0.8) | 34 | 1.0 (0.2) | 33 | 1.1 (0.4) | 28 | 1 (0) | 28 | 1 (0) |
| Reckless Behaviours | 38 | 2.0 (0.9) | 32 | 2.0 (0.8) | 27 | 1.9 (0.9) | 29 | 2 (1) | 34 | 1.5 (0.7) | 33 | 1.8 (0.9) | 28 | 1.6 (0.7) | 28 | 1.8 (0.8) |

*Note:* M = mean; SD = standard deviation; N = number of participants contributing to values. The first three variables were derived from the NIDA-Assist, the fourth variable was derived from the Daily Drinking Questionnaire, and the fifth through seventh variable was derived from the Risky, Impulsive, and Self-Destructive Questionnaire.

***Caution should be made because the mean values do not equate to raw score frequencies, refer to manuscript for original scoring and re-scaling to new groupings for ordinal scale.**
